# The patterns and associated factors of life-space in older patients at 6 months after percutaneous coronary intervention: a latent class analysis

**DOI:** 10.3389/fpubh.2025.1733092

**Published:** 2026-01-12

**Authors:** Shiyu Liang, Yanyan Zhang, Ruixue Tang, Weibo Lyu, Chenxi Zhu, Kangyao Cheng

**Affiliations:** 1 School of Nursing, Shanghai University of Traditional Chinese Medicine, Shanghai, China; 2 Shanghai Mental Health Center, Shanghai Jiao Tong University School of Medicine, Shanghai, China

**Keywords:** cardiac rehabilitation, older adults, latent class analysis, life-space, percutaneous coronary intervention

## Abstract

**Objectives:**

Life-space is a critical indicator of recovery in older patients after percutaneous coronary intervention (PCI). We sought to explore its latent classes at 6 months after PCI and to analyze the influence factors associated with different classes.

**Methods:**

A cross-sectional study was conducted at the cardiac research centers of three tertiary comprehensive hospitals located along the eastern coastal region of China. The study ultimately included 315 older patients who had undergone PCI as the participants. The patients’ life-space were assessed using the Life-space Assessment-China (LSA-C). Latent class analysis was employed to cluster the scores across different dimensions of life-space, and multinomial logistic regression was used to identify factors associating with the subgroups of life-space.

**Results:**

Three distinct subgroups were identified: daily activity group (C1, *n* = 94, 29.8%), community exercise group (C2, *n* = 156, 49.5%), and home maintenance group (C3, *n* = 65, 20.6%). Age (*p* = 0.004, OR = 0.865, 95% CI:0.783 ~ 0.955), diabetes history (*p* = 0.036, OR = 0.368, 95% CI: 0.144 ~ 0.937), depression (*p* = 0.003, OR = 1.253, 95% CI:1.079 ~ 1.455) only influence the transition of C3 to C1,whereas Duke activity status index (DASI) (*p* = 0.004, OR = 0.86, 95% CI: 0.783 ~ 0.955), self-efficacy (*p* = 0.030, OR = 0.695, 95% CI:0.501 ~ 0.964) influence the transition of both C2 and C3 to C1.

**Conclusion:**

Six months after PCI, the life-space of older patients can be classified into three subgroups. C1 exhibits the highest level of life-space, whereas the majority of patients fall into the lower-level subgroups (C2 and C3). Variations among these subgroups are associated with physiological and psychological factors. Identifying different subgroups of patients may provide new perspectives for medical professionals to individualized rehabilitation intervention protocols according, thereby alleviating restrictions of life-space and facilitate an effective transition from a home- and community-based setting to one cantered on routine daily activities.

## 1 Introduction

Coronary heart disease (CHD) is one of the most prevalent cardiovascular conditions globally (1). The global burden of CHD remains substantial according to the Estimates in 2025 (2), with an estimated 250 million individuals affected. As one of the most populous countries in the world, China has a significant CHD burden, with the 2021 prevalence reaching approximately 11.39 million cases, accounting for a significant proportion of the global total (3). Older populations constitute the primary demographic factor affected by CHD. According to the “China Cardiovascular Health and Disease Report 2024” (4), as of 2022, the mean age of hospitalized patients with CHD in China was 66.5 years, with 77.2% of patients aged within the range of 55–84 years. As a standard therapeutic approach for CHD, percutaneous coronary intervention (PCI) (5) enables revascularization and can significantly reduce the mortality. In 2023 alone, there are around 1.63 million of registered cases undergoing coronary artery disease interventions in China, supporting a significant expansion of the patient population (6). Nevertheless, post-PCI patients remain at risk for poor prognosis, underscoring the necessity for ongoing cardiac rehabilitation and follow-up care. Currently, in terms of well-established cardiac rehabilitation internationally, it typically consists of three phases, with the 6-month postoperative period representing a critical intermediate stage from Phase II to Phase III rehabilitation (7). As a crucial period for establishing sustained health behavior modifications, this stage is featured by an elevated risk of recurrent cardiovascular incidents. Older patients are particularly vulnerable during this stage, experiencing adverse cardiovascular events as well as various physical and psychological challenges, including immobility to various degrees (8), postoperative anxiety (9), decreased adherence to rehabilitation protocols (10), and compromised quality of life (11). Moreover, these patients may also contend with multimorbidity, presenting with atypical clinical symptoms and insidious disease progression, which complicates timely detection and intervention. Therefore, there is a significant challenge in the post-PCI health management of older patients at 6 months. The key for current research lies in the enhancement of scientific rigor and comprehensiveness of outpatient management. In order to maintain optimal recovery status and improving quality of life, it is important to strengthen follow-up, monitoring, and rehabilitation interventions during this period.

Unlike traditional physical function assessments such as Activities of Daily Living (ADL), Life space is an index that reflects a person’s mobility (12–14). Through the quantification of the scope, frequency, and independence of activities, this index can provide a comprehensive reflection of an individual’s activity capacity, willingness to participate, and environmental adaptability. It enables dynamic tracking of actual activity trajectories as it can reveal the interaction between intrinsic abilities and external environmental factors. In the context of health transitions among the older adults, life-space can provide numerous pieces of evidence regarding significant correlations with multidimensional indicators including overall health, daily functional capacity, somatic performance, and psychological wellbeing, thus offering unique advantages in assessing health status (15). As for the older adults, it can be utilized to predict disability (16), institutionalization (17), healthcare utilization (18), and mortality (19). In the cardiovascular domain, its multidimensional characteristics can facilitate a precise monitoring of the activity levels of patients during recovery (20, 21), revealing their self-management and interaction capabilities in real-life settings, and exposing potential hazards in daily activities (22). Eventually, it can contribute to the implementation of targeted rehabilitation interventions and enhancement of cardiac rehabilitation to benefit patient outcomes clinically. Beyond its predictive utility, an elevated level of life-space may indicate expanded activity range, increased activity frequency, and improved independence, which are facilitators of active rehabilitation, thereby supporting vascular health through multiple psychosocial and physiological pathways.

Currently, substantial older patients undergo PCI and may be accompanied by complex health issues. The postoperative six-month period represents a critical window for cardiac rehabilitation, necessitating intensified assessment and intervention. Life-space can be regarded as a crucial index for adjusting rehabilitation protocols during this phase, thereby playing a predictive and facilitative role in cardiac recovery. Nonetheless, there exists a paucity of research examining the life-space of post-PCI older patients and its heterogeneity, alongside a limited investigation into the primary factors that affect life-space interventions. Thus, the present study introduced and incorporated the conical model theory proposed by Webber et al. (23), a common tool in examining the determinants of life-space among community-dwelling older adults. This theory, encompassing six key elements: cognitive, psychosocial, physical, environmental, financial, and personal life history, can provide support for identifying potential influencing factors (24). This study was conducted to identify the subgroups of various dimensions of life-space at 6 months post-PCI by employing the latent class analysis (LCA), thereby gaining a novel perspective on individual differences in rehabilitation. Through the integration of the conical model theory, this study. Aims to explore the associated factors of different life-space subgroups of patients, providing target references for the identification and intervention of life space recovery in patients after PCI, with the expectation of further promoting cardiac rehabilitation.

## 2 Methods

### 2.1 Study design

This study was a cross-sectional design. Convenience sampling was utilized to select eligible older patients who had undergone PCI from March 2024 to June 2025 at three comprehensive class a hospitals’ cardiology centers along the eastern coast of China. Inclusion criteria encompassed: (i) patients aged over 60 years; (ii) had undergone PCI with successful procedural outcomes; (iii) had signed informed consent. Exclusion criteria included concurrent malignancies, severe hepatic or renal impairment, active infections or other significant organ failures; communication barriers; activity limitations; cognitive impairment.

### 2.2 Sample size

Based on conical model and literature reviews, we selected 27 possible influencing factors (23). According to Kendall’s cross-sectional sample size estimation method, the sample size should be 5 to 10 times the number of independent variables, considering a likely attrition rate of 10% and sampling error, the appropriate recommended sample size was range of 149 to 297 cases (25). Besides, to ensure the stability of the Latent Class Analysis (LCA) model (26), sample size exceeding 300 cases is recommended. In total, we successfully collected data from 315 participants.

### 2.3 Data collection

Under stable vital sign in post-PCI patients, we conducted the research. Prior to distributing questionnaires, we explained the research purpose to ensure informed consent. Some data of demographic and physical were collected through face-to-face interviews supplemented by patient’s clinical case documentation. Six months after PCI, telephone follow-ups were conducted to complete the LSA, DASI, PHQ-9, CDSES, and SRSS questionnaires. In total, among 329 CHD patients we approached, data from 315 participants were collected successfully, representing a response rate of 95.7%.

### 2.4 Instruments

#### 2.4.1 Life-space

The Chinese version of the Life-Space Assessment (LSA), adapted by Ji et al. (27), was used to evaluate life space levels. The LSA comprises five dimensions, with each dimension’s score derived from the product of distance travelled (5 levels, from bedroom only to beyond the neighborhood), frequency of travel (4 levels, from daily to < 1/week), and need for assistance (3 levels, with personal assistance / with equipment / none). Scoring is calculated as the product of the base score for each level, activity frequency, and independence level, with maximum scores of 8, 16, 24, 32, and 40 points for Levels 1 through 5, respectively, with scores below 60 indicating restricted life space. The Cronbach’s *α* coefficient is 0.803, with a test-retest reliability of 0.76 and good validity. Prior research14 indicates that assessment scores below the median of the scale range represent a restricted life-space; to simplify the model and facilitate interpretability, this study recorded the raw scores of the five dimensions of LSA-C into binary variables. Scores below the median were coded as 0, while scores above the median were coded as 1.

#### 2.4.2 Influence factors based on the conical theory framework

To comprehensively explore the potential influencing factors of life-space, this study adopts Weber’s conical theory framework. Based on the results of the expert panel meeting (28, 29), the measurement tools were selected accordingly. Since patients with cognitive impairments are excluded from our inclusion criteria, cognitive factors were not considered. The selection of independent variables focused on five dimensions: personal life history, economic status, psychological, physiological, and environmental factors, measured through relevant scales.

##### 2.4.2.1 Demographic factors (personal life history and financial)

Personal life history and economic status were unified under demographic factors. History of personal life includes gender, age, religious belief, marital status, employment status, occupation, and residence status. Economic variables encompass monthly household income.

##### 2.4.2.2 Physical factors

Including PCI method, number of PCI procedures, number of stent implantations, presence of common chronic diseases (e.g., hypertension, diabetes), and exercise endurance. Exercise endurance was assessed using the Chinese version Duke Activity Index (DASI): developed by Hlatky et al. (30), translated by Peng (31). It is a 12-item self-report scale covering daily activities such as mobility, household chores, walking, and recreational activities. Participants’ ability to complete each activity was evaluated, with total scores ranging from 0 to 58.2. The peak oxygen uptake (VO_2_peak) was estimated using the formula VO2peak (ml/kg) = 0.43 × DASI score + 9.6, serving as an indicator of exercise capacity—the higher the score, the greater the endurance. The scale has good reliability and validity among Chinese cardiovascular disease patients, Cronbach’s *α* coefficient is 0.803, with a test–retest reliability of 0.76 (31).

##### 2.4.2.3 Psychosocial factors

Including depression and self-efficacy. Depression was measured using the Patient Health Questionnaire-9 (PHQ-9) (32), a 9-item scale widely used for screening depression in cardiovascular patients. Each item is scored from 0 to 3 based on frequency, with total scores ranging from 0 to 27, the Cronbach’s *α* coefficient is 0.837; higher scores indicate more severe depression. Self-efficacy was evaluated using the Chronic Disease Self-Efficacy Scale (CDSES) (33), which consists of 6 items and two dimensions, can assess patients’ confidence in symptom management and disease-related tasks. Items are scored from 1 (not confident) to 10 (very confident). The mean score across six items reflects overall self-efficacy, with higher scores indicating a greater level of self-efficacy. The Cronbach’s *α* coefficient is 0.87.

##### 2.4.2.4 Environmental factors

Including surrounding environment (34), social (35) and familial environments (36). The surrounding environment was primarily evaluated based on accessibility and safety, including variables such as residence location post-discharge, proximity to common public venues (parks, schools, hospitals) within 15 min, and the presence and number of safety hazards within the life-space. Social and familial environments include social support and responsibility for family care. Social support was assessed using Social Support Rating Scale (SSRS) developed by Xiao (37), which encompasses three dimensions: subjective support (4 items, 8–32 points, *α* = 0.941), objective support (3 items, 1–22 points, *α* = 0.853), and utilization of support (3 items, 3–12 points, *α* = 0.916) (38). The total score ranges from 12 to 66, with higher scores indicating better social support.

### 2.5 Ethical considerations

This study has been approved by the Ethics Committee of Shanghai University of Traditional Chinese Medicine (approval number: 2024-1-16-10). All participants were informed of the study’s content and objectives prior to enrolment and provided written informed consent. All data collected in this research adhere to the principles of the Declaration of Helsinki, ensuring confidentiality and data integrity.

### 2.6 Statistical analysis

Latent class analysis (LCA) was performed with Mplus version 8.3 to identify subgroups of individuals based on life-space levels. The analysis began with a model specifying one latent class and incrementally increased the number of classes for model fitting estimation. Model fit was evaluated using the Akaike information criterion (AIC) (39), Bayesian information criterion (BIC) (40), and adjusted BIC (aBIC) (41), with lower values indicating better fit. The Lo–Mendell–Rubin adjusted likelihood ratio test (LMRT) and the bootstrapped likelihood ratio test (BLRT) were used to compare adjacent models (42); a *p*-value < 0.05 suggested a significantly improved fit with an additional class. Entropy was employed to assess classification accuracy, where higher values reflect better classification.

Statistical analyses of variables were conducted using SPSS version 26.0. Continuous variables are presented as mean ± standard deviation, and categorical variables as frequency and percentage. After identifying different latent classes via LCA, group comparisons were carried out using the *χ*^2^ test (or Fisher’s exact test for cell counts < 5) for categorical variables, ANOVA for continuous variables. Effect analysis is used to determine the true effect size of difference analysis *p*-value, whether there is a true difference and its magnitude, effect size of *χ*^2^ test is Cramér’s V, effect size of ANOVA is η^2^. According to the standards proposed by Cohen (43), the criteria for effect sizes in ANOVA are as follows: 0.01 indicates a small effect, 0.06 indicates a medium effect, and 0.14 indicates a large effect. For *χ*^2^ test, according to Cohen’s (44) guidelines for interpreting Cramér’s V coefficient, the standards are as follows: when df* = 2: 0.07 < V < 0.21 indicates a small effect, 0.21 < V < 0.35 indicates a medium effect, and V > 0.35 indicates a large effect, when df* = 3: 0.06 < V < 0.17 indicates a small effect, 0.17 < V < 0.29 indicates a medium effect, and V > 0.29 indicates a large effect, and so forth. Multinomial logistic regression was utilized to explore influence factors associated with latent class of life-space. To ensure the reliability of the regression model, a likelihood ratio test is conducted on the model, if *p* < 0.05 indicates that the model is effective as a whole. The explanatory ability of the model is achieved by referring to Pseudo R-Squared, which includes Cox & Snell *R*^2^, McFadden R, and Nagelkerke R. The values of these metrics range from 0 to 1, the larger the value, the better the model can explain the variation of the dependent variable (45). *p* < 0.05 was considered statistically significant.

## 3 Result

### 3.1 Descriptives of the sample

A total of 315 older patients undergoing PCI were included in the study. Demographically, the population was predominantly male (69.2%), primarily of Han ethnicity (98.7%), and mostly married (91.7%). Economically, the majority of participants reported a monthly household income ranging from 3,001 to 10,000 RMB (78.4%). Physically, hypertension prevalence was notably high at 72.7%. The proportion of patients undergoing emergency procedures was greater than those undergoing elective surgery (57.5% vs. 42.5%), with most receiving single-stent implantation (63.8%). The mean score of DASI was (34.30 ± 10.68). Psychologically, the mean scores of depressions and self-efficacy were (7.07 ± 3.82), (5.72 ± 1.78) respectively. Social and family environmentally, 40.3% of patients assumed caregiving responsibilities at home, and the mean score of social support was (41.24 ± 4.28).

### 3.2 Latent class of life-space

Four models were ultimately fitted in this study, with fit indices presented in Table 1. When the number of classes *n* > 3, the decline in model fit indices became less pronounced as the class number increased. Among the models evaluated, the three-class model showed the lowest AIC and aBIC, significant LMR and BLRT *p*-values (<0.05), and an entropy of 0.942, thus being selected as the optimal solution.

**Table 1:** Fit statistics for the latent class analysis.

Model	AIC	BIC	aBIC	Entropy	*P-value*		Mixing ration
					BLRT	LMR	
1	956.116	974.879	959.020	—	—	—	1.000
2	888.895	930.174	895.285	1.000	<0.001	<0.001	0.31429/0.68571
3	875.425	939.219	885.299	0.942	<0.001	<0.001	0.29841/0.49524/0.20635
4	880.555	966.864	893.914	0.866	<0.001	<0.001	0.19048/0.05714/0.00317/0.74921

AIC, akaike information criterion; BIC, Bayesian information criterion; aBIC, adjusted Bayesian information criterion; LMR, Lo–Mendell–Rubin; BLRT, bootstrapped likelihood ratio test.

**Figure 1:**
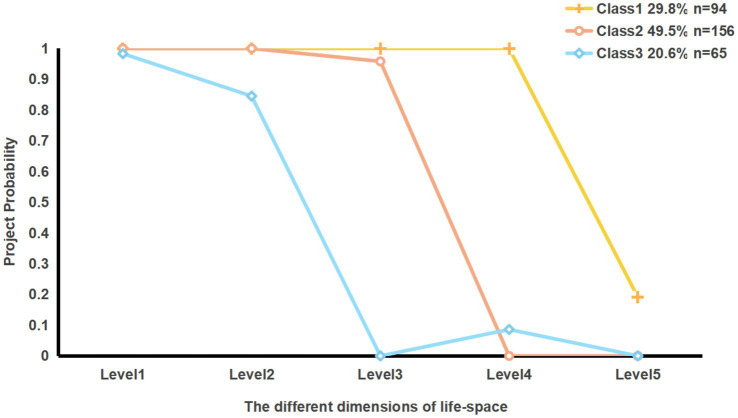
Level 1: a room in house other than the bedroom, Level 2: an area immediately outside home, Level 3: places in immediate neighborhood but beyond place of residence, Level 4: places beyond immediate neighborhood but within town, Level 5: outside of town.

Table 2 and Figure 1 show the restricted status of patients’ life-space, we named three latent classes according to their restriction levels. Class 1 (Daily Activity Group): Levels 1–4 are unrestricted; Level 5 indicates activities outside of town. Despite a high probability of restriction at Level 5, the mean scores across the three categories remain highest, indicating patients can generally perform routine activities; thus, designated as Daily Activity Group. Class 2 (Community Exercise Group): Levels 1–2 are unrestricted, with Level 3 representing intra-neighborhood activities, which have a restriction-free probability exceeding 90%, though some restrictions persist. Higher levels (4, 5) show lower scores and complete restrictions. Patients in this category primarily experience activity limitations within the community, hence classified as Community Exercise Group. Class 3(Home Maintenance Group): Only Levels 1–2, representing activities within the home and residential buildings, are unrestricted; Levels 3–5 outside the neighborhood are restricted, and all dimension scores in this class are the lowest among the three. The life-space is confined to the home, leading to the designation as the Home Maintenance Group. The proportions of these three classes are as follows: C1: 29.8% (*n* = 94), C2: 49.5% (*n* = 156), and C3: 20.6% (*n* = 65).

**Table 2:** Scores of life space among patients of different categories (*n* = 315, M ± SD).

Class/Level	Level1	Level2	Level3	Level4	Level5	Total score
Class1	8.00 ± 0.00	16.00 ± 0.00	19.95 ± 3.00	23.91 ± 1.71	16.65 ± 7.94	84.51 ± 9.48
Class2	7.94 ± 0.35	15.13 ± 1.85	19.38 ± 3.36	14.47 ± 3.13	9.54 ± 6.04	66.46 ± 11.05
Class3	7.72 ± 0.78	12.92 ± 3.20	10.94 ± 2.20	12.25 ± 5.21	5.88 ± 5.44	49.72 ± 11.15
Total	7.23 ± 0.44	14.91 ± 2.23	17.78 ± 4.65	16.79 ± 5.78	11.00 ± 7.66	68.39 ± 16.25
Reference range of scores	0 ~ 8	0 ~ 16	0 ~ 24	0 ~ 32	0 ~ 40	0 ~ 120

Class1, daily activities group; Class2, community exercise group; Class3, home maintenance group.

### 3.3 Univariate analysis of latent classes

Table 3 presents an overview of demographic characteristics, physical health status, psychosocial factors, and environmental conditions across different patient subgroups, highlighting notable differences. Chi-square tests and univariate analysis of variance reveal that variables such as age, educational level, current employment status, diabetes history, DASI, self-efficacy, depression, social support and responsibility for family care, the number of places reachable within 15 min, safety hazards in life-space, residential address post-discharge significantly influence the distribution of patients across different life-space subgroups (*p* < 0.05). Based on the effect size criteria, in the univariate analysis, the effect sizes of age, Educational level, employment status, social support, number of places reachable within 15 min, safety hazards in the life space, residential address post-discharge are small effects, while the effect sizes of the other significant variables are medium effects.

**Table 3:** Characteristics and Chi-square analysis of categorical variables results [*N* = 315, *n*(%)].

Variables	Type	Total sample *n*(%)/mean(SD)	CLASS			*χ*^2^/*F*	*p*	Cramer’s *v*/η^2^
			Class1 (*n* = 94)	Class2 (*n* = 156)	Class3 (*n* = 65)			
Gender	Male	218(69.2%)	71 (75.5%)	102 (65.4%)	45 (69.3%)	2.834	0.242	0.095
	Female	97 (30.8%)	23 (24.5%)	54 (34.6%)	20 (30.8%)			
Age	—	68.87 (5.94)	67.89 (6.42)	69.42 (5.52)	69.68 (5.90)	4.036^b^	0.014	0.027
BMI(kg/m^2^)	—	24.10 (2.57)	24.41 (2.65)	23.83 (2.46)	24.27 (2.69)	1.677^b^	0.189	0.011
Religious brief	None	266 (84.4%)	80 (85.1%)	133 (85.3%)	53 (81.5%)	0.528	0.768	0.041
	Yes	49 (15.6%)	14 (14.9%)	23 (14.7%)	12 (18.5%)			
Marital status	Other	26 (8.3%)	7 (7.4%)	14 (9.0%)	5 (7.7%)	0.215	0.898	0.026
	Married	289 (91.7%)	87 (92.6%)	142 (91.0%)	60 (92.3%)			
Educational level	Primary school /below	68 (21.6%)	18 (19.1%)	28 (17.9%)	22 (33.8%)	12.646	0.049	0.142
	Middle school	121 (38.4%)	41 (43.6%)	61 (39.1%)	19 (29.2%)			
	High school	89 (28.3%)	22 (23.4%)	46 (29.5%)	21 (32.3%)			
	University/college	37 (11.7%)	13 (13.8%)	21 (13.5%)	3 (4.6%)			
Occupation	Farmer	45 (14.3%)	8 (13.4%)	2 (15.4%)	13 (9.3%)	8.696^a^	0.369	0.117
	Worker	72 (22.9%)	19 (21.5%)	37 (23.7%)	16 (14.9%)			
	Civil servant	33 (10.5%)	9 (9.8%)	20 (16.3%)	4 (6.8%)			
	Teacher/medical staff	29 (9.2%)	11 (8.7%)	13 (14.4%)	5 (6.0%)			
	Company employee	136 (43.2%)	47 (40.6%)	62 (67.4%)	27 (28.1%)			
Employment status	Employed	49 (15.6%)	27 (28.7%)	18 (11.5%)	4 (6.2%)	18.698	<0.001	0.244
	Retired/other	266 (84.4%)	67 (71.3%)	138 (88.5%)	61 (93.8%)			
Monthly household income (RMB)	Below 3,000	30 (9.5%)	6 (6.4%)	16 (10.3%)	8 (12.3%)	8.536	0.201	0.116
	3,001–5,000	125 (39.7%)	35 (37.2%)	59 (37.8%)	31 (47.7%)			
	5,001–10,000	122 (38.7%)	36 (38.3%)	66 (42.3%)	20 (30.8%)			
	Above 10,000	38 (12.1%)	17 (18.1%)	15 (9.6%)	6 (9.2%)			
Living status	Living alone/other	106 (33.7%)	40 (42.6%)	47 (30.1%)	19 (29.2%)	4.772	0.092	0.123
	Living with spouse	209 (66.3%)	54 (57.4%)	109 (69.9%)	46 (70.8%)			
Hypertension	No	86 (27.3%)	31 (33.0%)	37 (23.7%)	18 (27.7%)	2.541	0.281	0.090
	Yes	229 (72.7%)	62 (67.0%)	119 (76.3%)	47 (72.3%)			
Diabetes history	No	198 (62.9%)	32 (34.0%)	48 (30.8%)	37 (56.9%)	13.994	0.001	0.211
	Yes	117 (37.1%)	62 (66.0%)	108 (69.2%)	28 (43.1%)			
Hyperlipidemia	No	257 (81.6%)	75 (79.8%)	130 (83.3%)	52 (80.0%)	0.628	0.730	0.045
	Yes	58 (18.4%)	19 (20.2%)	26 (16.7%)	13 (20.0%)			
Stroke	No	275 (87.3%)	79 (84.0%)	140 (89.7%)	56 (86.2%)	1.817	0.403	0.076
	Yes	40 (12.7%)	15 (16.0%)	16 (10.3%)	9 (13.8%)			
Smoking status	Never	151 (47.9%)	40 (42.6%)	78 (50.0%)	33 (50.8%)	6.347	0.175	0.100
	Quit	117 (37.1%)	33 (35.1%)	61 (39.1%)	23 (35.4%)			
	Still smoking	47 (14.9%)	21 (22.3%)	17 (10.9%)	9 (13.8%)			
Drinking status	Never	161 (51.1%)	39 (41.5%)	88 (56.4%)	34 (52.3%)	8.815	0.048	0.118
	Quit	97 (30.8%)	32 (34.0%)	41 (26.3%)	24 (36.9%)			
	Still drinking	57 (18.1%)	23 (24.5%)	27 (17.3%)	7 (10.8%)			
Surgical approach	Emergency	181 (57.5%)	60 (63.8%)	81 (51.9%)	40 (61.5%)	3.959	0.138	0.112
	Elective	134 (42.5%)	34 (36.2%)	75 (48.1%)	25 (38.5%)			
Number of stents implanted	1	201 (63.8%)	65 (69.1%)	100 (64.1%)	36 (55.4%)	3.343^a^	0.502	0.072
	2	101 (32.1%)	26 (27.7%)	49 (31.4%)	26 (40%)			
	3	13 (4.1%)	3 (0.02%)	7 (4.5%)	3 (4.6%)			
Number of PCI procedures	1	210 (66.7%)	61 (64.9%)	113 (44.6%)	36 (32.3%)	7.655	0.105	0.135
	2	79 (25.1%)	23 (24.5%)	35 (22.4%)	21 (44.9%)			
	over 3	26 (8.3%)	10 (10.6%)	8 (5.1%)	8 (12.3%)			
DASI	—	31.50 (15.99)	42.83 (14.66)	29.33 (14.19)	20.34 (11.34)	54.861^b^	<0.001	0.260
Self-efficacy	—	5.72 (1.78)	6.83 (1.44)	5.53 (1.68)	4.55 (1.55)	41.741^b^	<0.001	0.211
Depression	—	7.07 (3.82)	4.94 (2.97)	7.03 (3.31)	10.26 (3.90)	48.655^b^	<0.001	0.238
Social support	—	41.24 (4.28)	42.73 (3.66)	40.86 (4.45)	40.02 (4.18)	9.486^b^	<0.001	0.057
responsibility for family care	No	188 (59.7%)	48 (51.1%)	91 (58.3%)	49 (75.4%)	9.680	0.008	0.175
	Yes	127 (40.3%)	46 (48.9%)	65 (41.7%)	16 (24.6%)			
Number of places reachable within 15 min	7	219 (69.5%)	74 (78.7%)	105 (67.3%)	40 (61.5%)	6.072	0.048	0.139
	Less than 7	96 (30.5%)	20 (21.3%)	51 (32.7%)	25 (38.5%)			
Safety hazards in the life space	No	245 (77.8%)	79 (84.0%)	126 (80.8%)	40 (61.5%)	12.860	0.002	0.202
	Yes	70 (22.2%)	15 (16.0%)	30 (19.2%)	25 (38.5%)			
Residential address post-discharge	Urban	173 (54.9%)	49 (52.1%)	97 (62.2%)	27 (41.5%)	16.711^a^	0.002	0.165
	Township	24 (7.6%)	39 (41.5%)	43 (27.6%)	36 (55.4%)			
	Rural	118 (37.5%)	6 (6.4%)	16 (10.3%)	2 (3.1%)			

a Fisher’s Exact Test.

b ANOVA.

### 3.4 Multinomial logistic regression of latent classes

Using the three latent classes as dependent variables (C1: Daily Activities Group; C2: In-Community Exercise Group; C3: Home Maintenance Group), multinomial logistic regression analysis was conducted with variables showing statistical significance in univariate analysis as independent variables. Although our effect size analysis showed that some variables that were significant in the univariate comparison did not have large effect sizes, considering the combination with the conical theory model, we still included them in the regression analysis. The chi-square value of the likelihood ratio test was 190.176 (*p* < 0.001), which indicates that the model as a whole is significantly effective. The Nagelkerke R^2^ was 0.519, the McFadden pseudo-R^2^ was 0.292, and the Cox & Snell R^2^ was 0.453, suggesting moderate explanatory power of the model.

In the regression analysis, variables with smaller effect sizes were all excluded, except for age. The results revealed significant associations between different life-space subgroups and selected variables (Table 4). Compared to C1 (Daily Activities group), patients with reduced DASI (*p* = 0.01, OR = 0.95, 95% CI: 0.924 ~ 0.980), and lower self-efficacy (*p* = 0.022, OR = 0.771, 95% CI: 0.617 ~ 0.964) were more likely to belong to C2 (Community Exercise group). Conversely, younger age (*p* = 0.004, OR = 0.865, 95% CI:0.783 ~ 0.955), diabetes history (*p* = 0.036, OR = 0.368, 95% CI: 0.144 ~ 0.937), reduced DASI (*p* = 0.004, OR = 0.86, 95% CI: 0.783 ~ 0.955), lower self-efficacy (*p* = 0.030, OR = 0.695, 95% CI: 0.501 ~ 0.964), and higher depression (*p* = 0.003, OR = 1.253, 95% CI: 1.079 ~ 1.455) increased the likelihood of belonging to C3 (Home Maintenance group).

**Table 4:** Multiple regression analysis of latent class of life-space.

Variable		C2 *vs.* C1^a^			C3 *vs.* C1^a^		
		Estimate (SE)	OR (95% CI)	*p*	Estimate (SE)	OR (95% CI)	*p*
Age(years)	—	−0.033 (0.036)	0.968 (0.902 ~ 1.038)	0.358	−0.146 (0.051)	0.865 (0.783 ~ 0.955)	0.004
Diabetes history	No	−0.096 (0.346)	0.908 (0461 ~ 1.790)	0.781	−1.001 (0.477)	0.368 (0.144 ~ 0.937)	0.036
	Yes^a^	—	—		—	—	
DASI	—	−0.049 (0.015)	0.952 (0.924 ~ 0.980)	0.001	−0.101 (0.023)	0.904 (0.864 ~ 0.946)	<0.001
Self-efficacy	—	−0.260 (0.114)	0.771 (0.617 ~ 0.964)	0.022	−0.364 (0.167)	0.695 (0.501 ~ 0.964)	0.030
Depression	—	0.024 (0.058)	1.025 (0.914 ~ 1.149)	0.676	0.226 (0.076)	1.253 (1.079 ~ 1.455)	0.003

a Reference group.

C1, daily activity group; C2, in-community exercise group; C3, home maintenance group.

## 4 Discussion

### 4.1 Life-space of older patients at 6 months after PCI is at an intermediate level

The results of this study indicate that total mean scores of life-space of older patients at 6 months after PCI was 68.39 ± 16.25, which proved a generally intermediate level because of exceeded 60 points. However, analysis across various dimensions revealed a decline in life-space with the expansion of the activity range, accompanied by notable restrictions during broader activities such as traveling to places outside town. Consistent with previous research (22), this gradient phenomenon, from near to far, indicates the possibility of resuming small to medium-range domains daily activities in older patients after PCI, yet with great challenge in engaging in long-distance activities. In our speculation, it may be attributed to the objective physiological status and subjective willingness of patients. Patients who undergo early rehabilitation may be able to perform small to medium-range domains activities more effectively at 6 months post-discharge as they experience significant improvement in cardiac function and quality of life after intervention (46). However, activities beyond the neighborhood, involving larger physical exertion and greater cardiovascular demands, pose substantial challenges for these patients. Furthermore, there is a need to consider patients’ subjective willingness to engage in activities, in addition to their objective physiological status (47). For instance, for remote activities such as community outings, it has been reported that patients often require accompaniment from family members or caregivers (48). The level of familial support for outdoor excursions may have an impact on the patient’s subjective motivation, with some patients potentially avoiding such activities due to absence of companionship.

### 4.2 Latent classes of life-space among older patients at 6 months after PCI

Using LCA, this study identified three distinct latent subgroups of different dimensions life-space, including the daily activity group (C1), the community exercise group (C2), and the home maintenance group (C3). Patients in C1 group can perform activities outside the neighborhood relatively well, with only some restrictions within other districts of the town. For Chinese older adults, activities outside the community are mostly concentrated on medical appointments, routine shopping, and fee payments. Greater autonomy and social participation were observed in C1 group. Furthermore, the life-space in C2 group was mainly limited to within the neighborhood. In this range, older adults typically engage in leisure and rehabilitation activities (e.g., physical exercise, neighbourly visits, etc.), indicating a moderate level of life-space with room for improvement (49). In comparison, patients in C3 group were predominantly confined to their homes, with significantly reduced activity ranges below the average for patients with cardiovascular diseases (21). Moreover, these individuals also exhibited the lowest cardiopulmonary function and self-efficacy scores, but the highest depression levels, representing poorer rehabilitation outcomes. The rehabilitation outcome exemplified by patients in C1 group aligned more closely with the objective of cardiac rehabilitation, but approximately 70% of patients in this study were categorized into the C2 and C3 groups. Noticeably, older patients remain at elevated risk for recurrent CHD 6 months post-PCI (50). Therefore, Investigating the causes of varying life-space conditions and identifying strategies to enhance rehabilitation outcomes has become a primary focus of current research. In this context, our study also analyzes the associated factors influencing the differentiation among various life-space subgroups, with the objective of exploring possibility of facilitating adjustments within these subgroups and to support the efficacy of cardiac rehabilitation.

### 4.3 Influencing factors of latent class of life-space among older patients at 6 months after PCI

For the C3 group, age, diabetic history, and depression were independent factors affecting the transition of patients into the C1 group. Prior research (22) has documented the relationship of greater susceptibility to restricted life-space in older subjects. However, in our study, advanced-age patients demonstrated a higher likelihood of being classified into C1, which may be related to their frequent medical visits prompted by multimorbidity following PCI, which belongs to a passive expansion of life-space levels, rather than active engagement. As evidenced previously, the multimorbidity rate in the 60 ~ 69 age group was obviously lower than in those over 70 (51). Indirectly, compared to older counterparts, younger older patients have a lower outpatient medical consultation demand and may be influenced by their subjective activity intentions, favoring home-based maintenance. Therefore, it is critical to monitor older patients during assessment and follow-up for finding the real reason caused activity limitations, thereby facilitating a transition toward active engagement. However, the effect size of age in the univariate analysis is relatively small, which indicates that the impact of age on life-space remains a subject of debate, necessitating further verification. History of diabetes and depression were also key factors influencing the likelihood of classification into C3. Patients with diabetes experience impaired activity capacity as they are prone to develop complications such as motor dysfunction, diabetic foot ulcers, and medication-induced hypoglycemia. Approximately 45% of older diabetic patients had mobility impairments, (52) with an increased risk of falls. Additionally, patients usually occur recurrent symptoms (e.g., chest tightness, dull pain, and fatigue) within 6 months post-PCI. The physiological alterations after symptoms can precipitate psychological fluctuation (53), then progressively evolving anxiety and depressive states. Depression, in turn, can reduce physical activities and social participation (54). This interaction creates a bidirectional cycle of physiological and psychological impairment that may lead patients to avoid social and community involvement, further constricting patients’ life-space. Therefore, during six-month post-PCI, it is essential to conduct psychological assessments and interventions, in addition to addressing physical symptom management. Above conclusions and relevant literature, which underscores the requirement for linking physiological recovery, depression risk reduction, and life-space resumption. Medical staff may try to start by alleviating patients’ fears related to extensive activities to promote the formation of a positive cycle in all three aspects.

Simultaneously, DASI and self-efficacy were found to be common influencing factors for the transition from C2 and C3 to C1. DASI is a physiological indicator of cardiopulmonary function. In this study, lower scores on the index correlated with a higher likelihood of classification into C3 or C2 groups and greater propensity for categorizing into C3 than C2. Patients with reduced exercise tolerance tend to have limited activity ranges; and Tsai et al. (55) confirmed in their research that moderate to high-intensity activities occurred predominantly outside the home, and insufficient activity would exacerbate spatial confinement. In general, broader activity ranges are desirable goals during the six-month post-PCI rehabilitation, necessitating a foundational level of cardiopulmonary function. Concerning the mean DASI of 39.40 ± 10.75 points among Chinese patients with cardiovascular diseases reported previously (56), in our study, patients in C2 and C3 groups scored below this threshold, indicating significant functional deficits In general, broader activity ranges are desirable goals during the six-month post-PCI rehabilitation but necessitating a foundational level of cardiopulmonary function (21). Meanwhile, actively expanding the scope of life space is also conducive to improving cardiopulmonary function. This is indirectly that the three subgroups (C1, C2, and C3) can be transformed into each other. Chronic disease self-efficacy refers to the subjective confidence and perceived ability of patients to effectively control their condition and address disease-related challenges (57). In this study, patients who had lower scores were more likely to be categorized into C2 and C3 compared with C1. In other study (58) demonstrated positive correlations of self-efficacy with both mobility and activity levels; According to previous exploration, self-efficacy would exert a remarkable impact on health behaviors (59), quality of life, and psychological well-being among older patients with cardiovascular diseases. Patients exhibiting higher self-efficacy are more likely to engage in regular exercise and adhere to a healthy diet, which may subsequently affect their life-space. Therefore, self-efficacy may be a predictor of spatial heterogeneity in life space. In future care, perhaps the assessment of self-efficacy can be focused on in the early stage of rehabilitation, thereby having a certain grasp of the development of the patient’s future life space. In conclusion, both of them have certain influences on the heterogeneity of life space. The DASI score represents the patient’s physiological base level, while self-efficacy represents the patient’s intrinsic confidence in rehabilitation. During the subsequent cardiac rehabilitation process, this indicates that it is appropriate for medical staff to consider the rehabilitation after PCI from the perspective of balancing the patient’s physiological basis and psychological state, which can avoid overconfidence or excessive fear.

## 5 Limitations

The limitations of this study are primarily as follows: firstly, all samples were obtained from authoritative tertiary hospitals’ cardiac research centers located along the Chinese coast. Although these centers treat patients from across the country post-PCI, this may not guarantee the representativeness of the sample population. A broader national sampling of coronary heart disease patients is necessary to enhance the generalizability of the findings. Besides, considering the impact of sample size on the fluctuation of effect size, for example the conclusion regarding the age factor also requires further expansion of the sample size for verification. Secondly, existing research indicates that during the 6 to 12 months post-surgery (60, 61), patients’ health status fluctuates, with various complications arising, which are also critical for maintaining cardiac rehabilitation. Therefore, exploration of the longitudinal dimensions of life-space should be a priority in future studies.

## 6 Conclusion

In summary, this study investigated the life-space of 315 older patients at 6 months post-PCI. We found that the life-space of all patients at this period are predominantly at intermediate level and can be classified into three latent subgroups: Daily activity group (C1), Community exercise group (C2), and Home-based maintenance group (C3). Those subgroups differ significantly in terms of age, diabetes history, DASI score, self-efficacy, and depression. Based on our findings of this study, subgroup classification based on life space characteristics may help identify patients with distinct rehabilitation needs. Future research could explore the feasibility and effectiveness of developing dynamic, personalized rehabilitation programs tailored to these subgroups, with the aim of better supporting patients’ transition from home or community-based activities to their regular daily routines.

## Data Availability

The raw data supporting the conclusions of this article will be made available by the authors, without undue reservation.
